# Levothyroxine therapy in thyroidectomized patients: ongoing challenges and controversies

**DOI:** 10.3389/fendo.2025.1582734

**Published:** 2025-05-26

**Authors:** Leonardo Rossi, Marinunzia Paternoster, Mattia Cammarata, Sohail Bakkar, Paolo Miccoli

**Affiliations:** ^1^ Department of Surgical, Medical and Molecular Pathology and Critical Area, University of Pisa, Pisa, Italy; ^2^ Department of Surgery, Faculty of Medicine, The Hashemite University, Zarqa, Jordan

**Keywords:** levothyroxine therapy, thyroidectomy, dose adjustment, personalized therapy, heart risk, bone risk, quality of life, liquid levothyroxine

## Abstract

This mini-review provides an update on the challenges and controversies surrounding levothyroxine therapy in thyroidectomized patients, following an extensive review on dosing strategies and available formulations. Despite efforts to establish an ideal dosage adjustment method, achieving optimal thyroid hormone replacement remains complex due to interindividual variations in the hypothalamic–pituitary–thyroid axis and the pharmacokinetic and pharmacodynamic limitations of exogenous levothyroxine. Additionally, this review highlights the importance of evaluating the risk-benefit ratio of levothyroxine therapy, particularly in the setting of TSH suppression, focusing on its effects on quality of life, bone metabolism, and cardiac rhythm. Levothyroxine-induced subclinical hyperthyroidism may contribute to an increased risk of atrial fibrillation and alterations in bone mineral density, with implications that remain a subject of debate. Given the incomplete replication of endogenous thyroid hormone action by levothyroxine monotherapy, a tailored therapeutic approach is crucial. Despite ongoing research, the optimal management of thyroidectomized patients continues to be an open issue

## Introduction

Although approximately 5% of the population requires thyroxine therapy, dosage adjustment remains a significant clinical challenge (1). Patients who undergo total thyroidectomy require lifelong thyroxine replacement to prevent postoperative thyroid hormone deficiency. However, the pathophysiology of thyroidectomized patients is complex and influenced by multiple factors, particularly in specific subpopulations and those with comorbidities (2).

We previously published a review on levothyroxine (LT4) therapy in thyroidectomized patients, analyzing dosing strategies and different formulations reported in the literature (3). This mini-review provides a brief update on the topic, highlighting ongoing controversies and debated aspects of LT4 therapy in surgical patients.

## Levothyroxine therapy: difficulties in achieving the target

The European Thyroid Association (ETA) guidelines recommend adjusting LT4 therapy to maintain thyroid-stimulating hormone (TSH) levels within the 0.4−4.0 μIU/mL range (4). Moreover, in the context of surgical management of thyroid malignancies, the American Thyroid Association (ATA) guidelines recommend individualized TSH suppression targets based on the patient’s risk of recurrence and the presence of comorbidities (5). Specifically, a TSH level of <0.1 mU/L is advised for high-risk patients or those with a structural incomplete response. For intermediate-risk patients, or low-risk individuals with detectable serum thyroglobulin (Tg) or a biochemical incomplete response, as well as in high-risk patients with an excellent response to therapy, a TSH target of 0.1–0.5 mU/L is recommended. In contrast, low-risk patients with undetectable Tg levels should maintain TSH levels between 0.5–2.0 mU/L. Besides, the ATA guidelines underline that in patients at high risk of adverse effects on the heart and bone by TSH suppression therapy, the benefits should be weighed against the potential risks.

However, no single TSH value can universally indicate euthyroidism across all tissues, implicating an inherent limitation of LT4 monotherapy (6). A significant proportion of patients receiving LT4 therapy may exhibit TSH levels outside the established reference range, with overtreatment observed in nearly 17% and undertreatment in up to 10% of this population (7). Furthermore, a study investigating newly diagnosed hypothyroid patients initiating LT4 therapy reported a cumulative risk of 4.7% for overtreatment and 7.4% for undertreatment over 10 years. These observations highlight the ongoing challenge of determining the optimal LT4 dosage, a difficulty that is particularly pronounced in athyreotic patients (8). Additionally, many patients continue to experience symptoms suggestive of thyroid dysfunction despite having TSH and free thyroxine (fT4) levels within standard reference ranges (9). This observed discrepancy may arise from inter-individual variability in hypothalamic-pituitary-thyroid (HPT) axis set points, wherein optimal TSH and fT4 levels are more precisely defined at an individual level compared to broad population-based reference intervals. Accordingly, a recent investigation by Kuś et al. demonstrated that a polygenic score encompassing 59 genetic variants exhibited superior predictive power for individual TSH concentrations compared to fT4 or any assessed non-genetic factor. These findings suggest that individual genetic profiles hold promise for personalizing TSH reference ranges, potentially leading to substantial effects on LT4 prescriptions (10). Such challenges have fueled interest in advanced therapeutic approaches, leading to the development of a mathematical model known as Thyroid-SPOT (9). This algorithm provides a precise strategy for individualized euthyroid restoration and may assist clinicians in optimizing LT4 dose titration to address persistent dysthyroid symptoms in complex cases (9).

Previously, we extensively reviewed the primary LT4 dosing strategies reported in the literature, emphasizing that none have consistently achieved the target in all patients (3). In line with our prior findings, Valenzuela et al. (2) conducted a retrospective study on patients undergoing total thyroidectomy for benign conditions. Their analysis compared the accuracy of a novel Poisson regression model, which incorporates seven variables, against the conventional weight-based dosing approach for estimating LT4 requirements (11). While no statistically significant difference in predictive accuracy was observed between these models, the Poisson regression approach was associated with a lower incidence of LT4 overdosing (2).

Further complicating LT4 monotherapy, some researchers (12) have highlighted the physiological contribution of the thyroid to circulating free triiodothyronine (fT3) levels. In healthy individuals, approximately 20% of fT3 is directly secreted by the thyroid, while the remaining 80% originates from peripheral conversion of fT4. As a result, thyroidectomized patients may experience relative fT3 deficiency when relying solely on LT4 replacement, raising concerns about optimal therapeutic strategies. Addressing this issue, Ito et al. (13) conducted a retrospective analysis in 2022, evaluating the relationship between fT3 and fT4 levels in thyroidectomized patients receiving LT4 monotherapy. Their findings revealed that among patients with suppressed TSH, fT4 levels exceeded the reference range in 70.5% of cases, whereas fT3 remained within the reference range in 91.3% of individuals. Based on prior research, the authors suggested that patients with mildly suppressed TSH and normal fT3 levels were closest to euthyroidism when assessed through metabolic and symptomatic indicators (14, 15). These findings support the notion that monitoring fT3 levels, rather than solely relying on fT4, may improve management by ensuring a more balanced distribution of thyroid hormones within reference ranges (13). However, this remains a topic of considerable debate. Ettleson et al. (16) reported that the interplay between LT4, fT3, fT4, and TSH is complex and non-linear. While LT4 dose adjustments influence serum fT4 and TSH, their impact on fT3 levels is minimal and may not yield significant clinical benefits. Moreover, Jonklaas et al. conducted a prospective study which reported that after adequate adjustment of their LT4 doses, thyroidectomized patients had serum fT3 levels that were not significantly different from those before thyroidectomy and that normal fT3 levels were achieved with traditional LT4 therapy alone (17). These discrepancies align with previous animal studies, which suggest that thyroid hormone metabolism via type II deiodinase differs between the hypothalamus-pituitary axis and peripheral tissues (18). Overall, the ideal fT3 level required to optimize tissue-specific thyroid hormone action remains uncertain. Moreover, the mediators of T3—including physiological systems, genetic predispositions, and signaling pathways—are among the least understood factors influencing the metabolic effects of thyroidectomy (19).

Brun et al. (1) revisited the challenges of LT4 dose optimization in 2021 by developing a decision-support tool designed to model LT4 pharmacometrics. This tool facilitates individualized LT4 dosing based on serial fT4 and TSH measurements within the first two weeks post-surgery. The authors proposed that leveraging this computerized system could enable earlier dose adjustments (at two weeks rather than the conventional six to eight weeks), thereby improving treatment precision. Their findings indicated that the decision tool enhanced target TSH achievement rates in patients with goiter or thyroid cancer, although it did not improve dose adjustments in thyrotoxic individuals. Furthermore, it shortened dose optimization time by 40 days in thyroid cancer patients and 58 days in those with goiter, leading to a reduction in both follow-up visits and the need for repeated blood tests (1).

Additional factors may also contribute to the difficulty in achieving optimal LT4 therapy in thyroidectomized patients. For instance, studies have shown that in patients with differentiated thyroid cancer (DTC), fT4 increases following radioactive iodine (RAI) therapy are attenuated during the first month post-treatment. This phenomenon may theoretically be linked to gastrointestinal mucosal disruption induced by RAI therapy, potentially affecting LT4 absorption (20).

## New levothyroxine formulations

In recent years, new LT4 formulations have been introduced to enhance the effectiveness of LT4 therapy. One of these is a liquid formulation, where LT4 is dissolved in glycerol, purified water, and ethanol. Another is a soft gel capsule, in which the active compound is solubilized in glycerol and enclosed within a gelatin shell (21). Unlike conventional tablets, the liquid formulation does not require an acidic gastric environment for absorption, while the soft gel capsule rapidly dissolves in gastric acid, leading to faster absorption of LT4 compared to tablets (22).

Liquid LT4 offers a significant advantage for patients who have difficulties in swallowing solid dosage forms and has recently been associated with improved quality of life (23).

Additionally, studies suggest that liquid LT4 minimizes the absorption variability caused by food and coffee, bypasses malabsorption linked to increased gastric pH, and avoids absorption issues in patients who have undergone bariatric surgery. Conversely, although the effectiveness of soft gel LT4 has been investigated in a limited number of clinical studies, the results have been encouraging (22, 24, 25).

Nonetheless, following the prospective study by Fallahi et al. (26), which documented a better control of TSH in thyroidectomized patients under a liquid LT4 regimen over tablet form, research on alternative LT4 formulations in thyroidectomized patients remains scarce. Recent literature primarily consists of reviews summarizing previous studies on this topic (27–29). However, long-term adherence to LT4 therapy has been examined by Bocale et al. (30). In a study involving 106 thyroidectomized patients receiving LT4 replacement therapy in either liquid or solid form, the authors observed that the overall Medication Adherence Questionnaire score was significantly better in those treated with the liquid formulation. Furthermore, patients taking LT4 tablets were more likely to forget their medication and demonstrated greater inconsistency in adherence compared to those on liquid LT4 therapy (30).

## Effects of levothyroxine therapy on quality of life after thyroidectomy

Health-related quality of life (HRQoL) is a key concern for patients undergoing thyroid surgery, particularly those with DTC, given the excellent prognosis of this malignancy. Evaluating the impact of levothyroxine therapy on their quality of life is an area of growing interest in patient-centered outcomes research.

Altuntas et al. performed a study on a cohort of 191 DTC patients and 79 healthy controls to assess the psychological and sleep-related impact of long-term LT4 therapy and varying degrees of TSH suppression. Patients were stratified into three groups based on TSH levels: suppressed (<0.1 μIU/mL), mildly suppressed (0.11–0.49 μIU/mL), and low-normal (0.5–2.0 μIU/mL). The study demonstrated a higher prevalence of depression, anxiety disorders, and sleep disturbances among patients with DTC, with these symptoms being more pronounced in individuals with suppressed TSH levels. An inverse correlation was observed between TSH levels and the severity of psychological and sleep-related symptoms, while a positive correlation was found with the duration of LT4 therapy. These findings highlight the importance of avoiding unnecessary TSH suppression in the management of patients with DTC (31).

In 2022, Yaniv et al. (6) conducted a study to assess the quality of life in patients who had undergone thyroidectomy in relation to thyroid hormone replacement therapy. Among 160 participants, 107 were receiving levothyroxine therapy, regardless of the extent of surgery. Patients on levothyroxine reported significantly higher scores for fatigue, emotional distress, cosmetic concerns, and overall self-assessment compared to those who did not require hormone replacement. Additionally, there was a trend toward higher depression scores in patients receiving levothyroxine therapy (p = 0.06) (6). Consistent with previous findings (32), levothyroxine therapy was associated with worse emotional well-being, even in patients with normal TSH levels, suggesting a direct impact of the medication itself (6).

Despite these studies, the topic remains a matter of debate, and several aspects require further clarification. Recently, Monzani et al. (33) investigated the influence of different stages of levothyroxine therapy (withdrawal, full or mild TSH suppression, or replacement) on the quality of life of DTC patients. The study found that the poorest quality of life was observed in hypothyroid patients who had discontinued levothyroxine before RAI therapy. This outcome may have both biological and psychological explanations, with the latter potentially linked to the recent cancer diagnosis and concerns about health status and radioiodine treatment. Conversely, patients on suppressive levothyroxine therapy reported better quality-of-life scores compared to those on standard replacement therapy. This result may be only partially explained by the subjective feeling of being treated for cancer; furthermore, it leads to the conclusion that the normalization of TSH level may not be enough to achieve a good quality of life in some patients (33). However, the administration of supraphysiological doses of levothyroxine in DTC patients requires careful evaluation, as overtreatment carries potential health risks (34).

Patients undergoing thyroid lobectomy generally experience better postoperative quality of life than those undergoing total thyroidectomy, at least in the short term. However, some decline in quality of life may still occur even after less extensive surgery (35). A multicenter prospective randomized study by Lee et al. on 669 DTC patients undergoing thyroid lobectomy found that those with high TSH levels had significantly better physical domain scores three months postoperatively than those with low TSH levels. However, no significant differences were observed in overall HRQoL (36).

A special mention should be reserved for the impact of thyroid function on cognitive decline. A recent review reported that despite growing interest in the potential role of thyroid dysfunction as a modifiable risk factor for cognitive decline in the aging population, current evidence remains inconclusive, and no definitive recommendation can be made to inform clinical practice (37).

## Effects of levothyroxine therapy on cardiac rhythm after thyroidectomy

Cardiac arrhythmias are defined as irregular heartbeats, and their association with hyperthyroidism has been well established (38, 39). According to standard guidelines (5), the treatment of DTC may include postoperative full or partial TSH suppression to reduce the risk of recurrence. However, this approach leads to a state of exogenous subclinical hyperthyroidism (40), which may increase the risk of arrhythmias. This concern is particularly relevant in elderly patients, who often have additional cardiovascular risk factors.

Gong et al. (41) conducted a large population-based study using administrative healthcare databases from Ontario, Canada. The authors identified adults over the age of 66 without a prior history of atrial fibrillation (AF) who had received at least one levothyroxine prescription between 2007 and 2016 and subsequently developed AF. These cases were matched with up to five control patients without AF during the same period. The study found a statistically significant association between high and moderate levothyroxine exposure and AF risk. Specifically, levothyroxine doses exceeding 0.075 mg/day were linked to a higher incidence of AF compared to lower doses (41). Notably, this increased AF risk associated with higher levothyroxine doses is particularly relevant for thyroidectomized patients for DTC, who are more likely to be on full replacement therapy and thus receive higher daily doses of levothyroxine.

Paroxysmal, sustained, or permanent settings of AF lead to a substantial clinical burden and negatively impact patients’ quality of life. AF is the most common cardiac complication of hyperthyroidism and levothyroxine therapy-induced thyrotoxicosis, occurring in up to 15% of hyperthyroid patients compared to 4% in the general population (42). Additionally, Liu et al., in a study on 271 DTC patients, reported that those with suppressed TSH exhibited increased sympathetic activity and reduced vagal tone compared to euthyroid patients, resulting in greater heterogeneity in ventricular recovery time. The authors concluded that TSH suppression may influence heart rate variability and ventricular repolarization (43). Similarly, Celik et al. recently investigated the effects of different TSH suppression levels on cardiac electrophysiology in DTC patients. Although no statistically significant difference was observed between patients receiving suppressive versus replacement levothyroxine therapy, QT dispersion tended to increase as TSH suppression intensified (44).

However, the impact of TSH suppression on cardiac rhythm remains a topic of debate. Kaziród-Wolski et al. conducted a prospective study on 73 women with DTC undergoing levothyroxine suppression therapy (48 fully suppressed, 25 partially suppressed) and compared them to 25 healthy women (40). The study found no significant differences between groups in terms of maximum, average, or minimum heart rate, nor the incidence of cardiac arrhythmias. The authors concluded that maintaining fT3 levels within the normal range prevents clinically significant changes in heart rate or arrhythmia development in patients receiving suppressive levothyroxine therapy following thyroidectomy for DTC (40). Nevertheless, levothyroxine therapy should be administered with caution in patients with pre-existing cardiovascular disease (42).

While AF is the most frequently reported arrhythmia associated with thyrotoxicosis, ventricular arrhythmias may also be a potential adverse effect of levothyroxine suppression therapy. In 2021, Hepsen et al. (45) evaluated electrocardiographic (ECG) predictors of ventricular arrhythmia in DTC patients receiving levothyroxine suppression therapy. These patients were compared to 100 randomly selected healthy volunteers attending a cardiology outpatient clinic for routine check-ups. The study found that ECG indicators of ventricular arrhythmia were significantly more prevalent in DTC patients under levothyroxine suppression therapy. These findings suggest that clinicians should be vigilant about the potential cardiac risks associated with suppressive levothyroxine therapy (45).

## Effects of levothyroxine therapy on bone after thyroidectomy

Given the excellent prognosis of thyroid cancer, it is crucial to carefully evaluate the risk-benefit ratio and the potential side effects of TSH suppression therapy. Supraphysiological doses of levothyroxine may have adverse effects on bone health (46). While endogenous hyperthyroidism is known to increase the risk of osteoporosis and osteoporotic fractures (47), the impact of TSH suppression due to levothyroxine therapy remains a subject of debate (48). Previous studies have suggested a possible association between chronic TSH suppression therapy and reduced bone mineral density (BMD) in postmenopausal women with DTC, whereas no such correlation has been observed in premenopausal women or men (49, 50).

A recent meta-analysis by Kwak et al. (48), including 1127 patients (426 postmenopausal women with DTC on levothyroxine suppression therapy and 701 controls), reported that stringent TSH suppression (TSH <0.10 mIU/L), but not moderate suppression (TSH 0.10–0.49 mIU/L), was associated with a decline in postoperative lumbar spine BMD. However, no significant association was found between TSH suppression and femoral neck BMD in postmenopausal women. These discrepancies may be explained by the different bone compositions: the lumbar spine (the most frequent site of osteoporotic fracture) consists primarily of trabecular bone, which is more susceptible to osteoporotic changes, whereas the femoral neck is largely composed of cortical bone, which undergoes a more gradual mineral loss over time. Moreover, lumbar spine mineral content is particularly affected by menopause, whereas femoral neck BMD declines progressively throughout life.

In 2021, Sousa et al. (51) conducted a prospective study to evaluate trabecular bone score (TBS) – a textural index that provides an indirect assessment of bone microarchitecture and has been shown to predict incident major osteoporotic fractures (52) - in women with DTC on long-term levothyroxine therapy. Patients were divided into two groups based on the type of levothyroxine regimen: suppressive therapy or replacement therapy. While no statistically significant differences were observed between the groups, TBS was reduced in more than 50% of postmenopausal women with thyroid cancer receiving levothyroxine therapy. This suggests that even low-normal TSH levels, not just suppressed TSH, may contribute to impaired bone microarchitecture in postmenopausal women (51). Similarly, Jia et al. reported a 0.27-fold decline in BMD T-score and an increased fracture risk in patients receiving TSH suppression after thyroidectomy (53). Comparable results were obtained by Lin et al. (54).

However, the relationship between TSH suppression therapy and its effects on bone metabolism remains unclear, with previous studies yielding conflicting results (51, 55). A recent prospective controlled study by Wang et al. found that one year of postoperative TSH suppression therapy did not significantly affect BMD in men, premenopausal women, or postmenopausal women with DTC (56). Consistently, Heijckmann et al. (57) reported that patients with DTC undergoing long-term suppressive levothyroxine therapy did not show an increased risk of low BMD or vertebral fractures, particularly when treated with relatively low doses of levothyroxine.

It is worth noting that the debated skeletal effects of TSH suppression therapy have traditionally been attributed to the relative increase in circulating thyroid hormones. However, the identification of TSH receptors on murine osteoblasts and osteoclasts (55, 58) suggests that TSH itself may play a direct role in bone metabolism, independent of thyroid hormone levels. This remains an area of ongoing research and debate.

## Conclusions

Levothyroxine therapy in thyroidectomized patients remains a topic of ongoing discussion, requiring careful consideration by clinicians. Currently, a personalized approach that considers multiple factors is the best strategy for optimizing management. The physiological effects of thyroid hormones on peripheral tissues are not fully replicated by exogenous levothyroxine administration due to both pharmacokinetic and pharmacodynamic limitations, as well as individual variations in the HPT axis, which may not be fully reflected in standard thyroid function tests.

Furthermore, levothyroxine therapy must be carefully tailored based on the underlying thyroid condition (benign or malignant) and the patient’s comorbidities, particularly regarding its impact on bone health and cardiac rhythm. Moreover, its effects on quality of life should also be taken into account. In this context, the latest ATA guidelines (5), considering the excellent outcomes in patients with DTC and the resulting treatment de-escalation (such as less extensive surgery—e.g., lobectomy—or even active surveillance, and reduced use of RAI therapy), recommend balancing the potential benefits of suppressive LT4 therapy against its associated risks. Given all these complexities, the discussion surrounding the optimal therapeutic approach for thyroidectomized patients remains open.
